# Improved Prediction of Harmful Algal Blooms in Four Major South Korea’s Rivers Using Deep Learning Models

**DOI:** 10.3390/ijerph15071322

**Published:** 2018-06-24

**Authors:** Sangmok Lee, Donghyun Lee

**Affiliations:** Department of Business Administration, Korea Polytechnic University, 237, Sangidaehak-ro, Siheung-si, Gyeonggi-do 15073, Korea; tkdahr1331@gmail.com

**Keywords:** algal blooms, deep learning, artificial intelligence, chlorophyll-a, LSTM

## Abstract

Harmful algal blooms are an annual phenomenon that cause environmental damage, economic losses, and disease outbreaks. A fundamental solution to this problem is still lacking, thus, the best option for counteracting the effects of algal blooms is to improve advance warnings (predictions). However, existing physical prediction models have difficulties setting a clear coefficient indicating the relationship between each factor when predicting algal blooms, and many variable data sources are required for the analysis. These limitations are accompanied by high time and economic costs. Meanwhile, artificial intelligence and deep learning methods have become increasingly common in scientific research; attempts to apply the long short-term memory (LSTM) model to environmental research problems are increasing because the LSTM model exhibits good performance for time-series data prediction. However, few studies have applied deep learning models or LSTM to algal bloom prediction, especially in South Korea, where algal blooms occur annually. Therefore, we employed the LSTM model for algal bloom prediction in four major rivers of South Korea. We conducted short-term (one week) predictions by employing regression analysis and deep learning techniques on a newly constructed water quality and quantity dataset drawn from 16 dammed pools on the rivers. Three deep learning models (multilayer perceptron, MLP; recurrent neural network, RNN; and long short-term memory, LSTM) were used to predict chlorophyll-a, a recognized proxy for algal activity. The results were compared to those from OLS (ordinary least square) regression analysis and actual data based on the root mean square error (RSME). The LSTM model showed the highest prediction rate for harmful algal blooms and all deep learning models out-performed the OLS regression analysis. Our results reveal the potential for predicting algal blooms using LSTM and deep learning.

## 1. Introduction

### 1.1. Overview

Harmful algal blooms are a phenomenon in which the water in rivers and lakes turns dark green because of excessive algal growth [1]. They can affect areas used as water sources, potentially causing harm to humans and animal, e.g., acute or chronic liver damage when the contaminated water is ingested [2,3]. Moreover, water contaminated by harmful algal blooms looks unappealing and contains a water-soluble neurotoxic component [4]. The effects of harmful algal blooms on rivers and lakes have been experienced and reported worldwide, including the large-scale death of fish [5]. For example, the 11 US health and environment departments funded by the National Center for Environmental Health (NCEH) received a total of 4534 reports on animal disease outbreaks, deaths, and human diseases related to the occurrence of harmful algal blooms from 2007 to 2011 [6], with damages in the US alone estimated at more than 2.2 billion dollars per annum [7]. Therefore, harmful algal blooms are of worldwide concern due to their potential for human and environmental harm along with economic losses [8].

Although environmental authorities worldwide are taking precautions to eliminate such blooms, finding a fundamental solution to the recurring problem is difficult. In South Korea, for instance, the Ministry of Environment sends out alerts via local algae warning and water quality forecasting systems. The algae warning system is based on field survey data for 28 major water sources, lakes, and rivers. The water quality forecasting system is based on actual data such as water temperature and weather observations for 16 dammed pools on four major rivers. Such systems measure the concentrations of cyanobacteria (actual) and chlorophyll-a (predicted) in the water.

Models traditionally used for predicting water quality include QUAL2E (United States Environmental Protection Agency, Washington, WA, USA), CE-QUAL-W2 (2-D Hydrodynamic Water Quality Model, The U.S. Army Corps of Engineers, Washington, WA, USA) and others. As such models are based on actual measurements, variations and changes can be checked through mathematical calculations of each element [9]. However, it is expensive and time-consuming to build and operate these models. It is also difficult to set a clear coefficient indicating the relationship between each factor when using such physical models.

Although QUAL2E has 15 water quality parameters that can be simulated, there are some limitations. For example, the measured value for an increase in biological oxygen demand (BOD) is limited due to the production and death of algae, so that it is not possible to simulate a large-scale river [10]. CE-QUAL-W2 considers water level, flow, water temperature, and many other water quality factors while also considering the total amount of sediment, ammonia-nitrogen, and phosphate-phosphorus. In other words, this model needs to calculate ~20 derived components including pH and carbonate species [11]. Thus, for these models to be effective, many variable data sources are required for analysis, which can limit the application of the model, so recent research has focused on the use of machine learning techniques that can overcome these limitations [12].

Machine learning (and its sub-method, deep learning) can analyze and learn a vast amount of untapped big data, extracting important patterns from the datasets and providing insight into specific research questions or problems [13]. In addition, deep learning’s capacity to determine the most important features can efficiently provide data scientists with concise and reliable analysis results. Consequently, deep learning has improved research techniques dramatically in fields such as speech recognition and genetics [14], and its use in various fields is increasing. For example, Song et al. [15] used deep learning to predict gastrointestinal infection rates using an environmental context because it is difficult to deal with such complex predictive problems due to the many influential indicators and unknown probabilistic relationships between indicators and disease. Li et al. [16] used the long short-term memory (LSTM) model to predict the spatio-temporal patterns of fine particulate (PM_2.5_) pollution in China. Previous studies have revealed an increase in the use of deep learning models in environmental studies. In the study of rainfall–runoff modeling, the MLP model was compared with a traditional statistical model, and showed better performance [17]. Recently, the LSTM model has been applied to time-series prediction, for example, wind power prediction [18] and PM_2.5_ pollution risk prediction [16]. For PM_2.5_ prediction, the model showed higher prediction precision than other time-series prediction models (support vector machine, SVM; and autoregressive moving average, ARMA) and a traditional neural network for feature representation (time delay neural network, TDNN) [16].

In South Korea, although algal blooms occur consistently [19,20,21], the deep learning model has not often been used in water quality research, despite its proven performance. Therefore, in this study, we used machine learning (specifically the deep learning method) to build a water quality prediction model for harmful algal blooms. Existing deep learning models include the multi-layer perceptron (MLP) network and the Elman neural network, a type of recurrent neural network (RNN). We also employed the LSTM model, which is optimized to handle time series data better than other models [22]. For example, when the amount of data increases in an RNN model, past data values of the algorithm are lost at a high rate through the calculation process. However, the LSTM model solves this problem and minimizes such data loss [23], potentially allowing it to more accurately estimate the time of algal bloom occurrence and help prevent them, reducing possible damage. Applying this deep learning model to water quality prediction can therefore reduce the loss of time and money related to harmful algal blooms, aiding research development in this field and contributing to a fundamental solution to this environmental problem.

In this study, we aimed to build a more precise prediction model that would facilitate pre-emptive action to prevent or mitigate the effects of harmful algal blooms in South Korea. First, we selected variables affecting the occurrence of harmful algal blooms through a regression analysis of water quality and quantity data provided by the South Korean Ministry of Environment and the Ministry of Land, Infrastructure and Transport. Second, we constructed and compared the MLP, RNN, and LSTM deep learning models. Data collected by the Ministry of Environment and local units were used for the analysis to ascertain both dependent variables and independent variables. Through this process, we found that deep learning models generated more accurate predictions than traditional evaluation models.

### 1.2. Literature Review

Various factors can cause harmful algal blooms, such as an increase in nutrients from an influx of anthropogenic contaminants produced by households, factories, farmland, or other sources [24]. These nutrient increases create a favorable environment for algal growth [25]; other contributing factors include water temperature and insolation [26]. Cyanobacteria, which are the main cause of harmful algal blooms, are known to reproduce optimally at a water temperature of 25 °C [27]. The flow and circulation of water can also contribute to algal blooms [28]; when water circulation is inadequate, algae remain in the upper layer of the water after bloom occurrence, leading to abnormal residence times and the reoccurrence and rapid spread of algal blooms [29]. However, sufficient flow and circulation creates an environment in which algae cannot breed in one place, reducing the occurrence of blooms [30].

In other words, algal blooms occur because of eutrophication [31]. For example, Florida Bay in the southwestern United States is disturbed frequently by large and dense algal blooms resulting from the sediments and nutrients in the water. Although eutrophication here is most likely caused by the algal biomass, it is difficult to measure this biomass directly, so chlorophyll-a is used as a measure of eutrophication. Chlorophyll-a is an indicator of phytoplankton, is sensitive to excessive nutrients, and can be monitored continuously. In addition, an appropriate limit relevant to chlorophyll-a has been set, which can be utilized as an indicator of water pollution. Similarly, a factor analysis on the Taihu River (China) determined the cause of algal occurrence by using chlorophyll-a as an indicator [32], showing that temperature, pH, total nitrogen (TN), total phosphorus (TP), and other environmental factors (as well as anthropogenic pollution) affected the growth of algae.

In addition, changes in the chlorophyll-a concentration in relation to water quantity and flow rate are also factors in the formation of algal blooms, as shown by an analysis of water level fluctuations in Xiangxi Bay (China) [28]. These results suggested that raising the water level could modulate the occurrence of harmful algal blooms, as the eutrophication stratum became vertically blended when more water was introduced, reducing the time the algae remained on the surface. In this way, algal propagation can be reduced because of dilution and dispersion of the nutrients.

In summary, increases in chlorophyll-a can be caused by several significant water quantity and quality factors. In this study, we used chlorophyll-a as an indicator for water quality (and thus algal blooms) and considered factors such as temperature, pH, biochemical oxygen demand (BOD), COD, DO, cyanobacteria, water level, and pondage in combination with water quality and quantity data in our analysis of the causes of harmful algal blooms. Various studies have demonstrated the use of deep learning models to predict water quality, such as by predicting dissolved oxygen (DO), TN, and TP concentrations using an Elman neural network [33]. However, as described above, we used an LSTM model that can better analyze these data and potentially reduce or prevent the damage caused by harmful algal blooms by achieving more accurate predictions of their occurrence.

This paper is structured as follows: Section 2 presents the scope of the research and the necessary background information on the MLP, RNN, and LSTM models, as well as a description of the variables used in the experiments. Section 3 explains the setup of the experiments, and presents and discusses the results. Section 4 provides the conclusions of this study.

## 2. Methodology

### 2.1. Scope and Composition of Research

**Figure 1:**
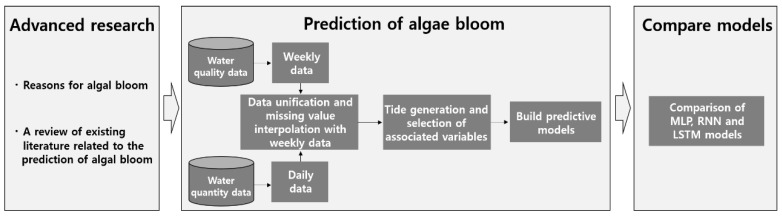
Research framework showing the three stages used to compare deep learning models.

In this study, we aimed to identify the cause of harmful algal blooms and construct a suitable prediction model to facilitate preemptive action. We used ordinary least square (OLS) regression with the MLP, RNN, and LSTM models to build our prediction model. The study’s three-stage framework is shown in Figure 1.

First, we investigated the measurement criteria and causative factors for harmful algal blooms and assessed previous attempts to use machine learning models for water quality prediction. Second, we constructed our dataset by combining water quality and quantity data, then performed OLS regression analysis, which is typically used in empirical analysis. The analysis determined the influence of the independent variable on the dependent variable and whether it was positive or negative through correlation coefficients [34]. The results identifies the variables with the potential to have a significant effect on chlorophyll-a prediction. In addition, we used the combined water quality and quantity dataset to compare the deep learning model with the predicted values. Finally, we constructed a combined model that could offer one-week predictions, using data from 16 dammed pools on 4 major river basins and the MLP, RNN, and LSTM models.

For the effective prevention of algal blooms, we built a model that could predict results one-week in advance. The one-week prediction period was chosen with reference to past research indicating that harmful algal blooms are characterized by rapid breeding when the environmental requirements are met [35]. As a comparative indicator, the accuracy of prediction between the models was examined by comparing the RMSE values commonly used as a measure of model performance [36]. It is determined by the difference between the predicted and actual values measured by the model and is a good indicator of the average error [17,37,38]. The final goal was a deep learning model that could predict harmful algal blooms in the four major rivers of South Korea.

### 2.2. Analytical Model

We used OLS linear regression analysis to identify the factors that could contribute to harmful algal blooms by analyzing their effects on chlorophyll-a; this approach determines the most basic linear relationship between dependent and independent variables. The variables for deep learning analysis and multiple regression analysis were chosen by backwards elimination to select a meaningful value that could satisfy the assumption in multiple regression analysis. The multicollinearity verification results for these variables were all lower than the variance inflation factor (VIF) of 10, and were also used in the MLP, RNN, and LSTM models. The formula for the regression analysis was:

(1) $$Chlorophyll–a=\beta_{0}+\beta_{1}\left(temperature\right)+\beta_{2}\left(pH\right)+\cdots +\beta_{8}\left(pondage\right)+\varepsilon_{i},\;i=1,\ldots n$$

Regression analysis showed that the cyanobacteria had larger standard deviations than the other variables. If these data were used as-is, the maximum-minimum normalization would be performed on all selected variables, as data with large values could have a more significant effect on the result than other data. The maximum-minimum normalization transformed the distribution of the values from 0 to 1, using the maximum and minimum values of the data.

We compared and analyzed the deep learning model based on the RNN and LSTM models (that mostly use sequential data) and the MLP model. The nonlinear activation function was used in the MLP, except for the input layer.

In the MLP model, after performing the feed-forward calculation to determine the weight for each node, the error was reduced by learning the optimal weight and bias through the back-propagation algorithm, which reduced the error by sending the error between the predicted value and the actual value of the error backward [39]. Through back-propagation, the neuron weights are updated by a gradient descent method in response to errors between neural networks to determine the weights that minimize errors. This has the advantage of adjusting parts that are difficult to adjust using human intuition in a complex system [40].

The existing RNN model was used to develop the LSTM model. Comparing the RNN, LSTM, and MLP models, the MLP model ignored the time sequence and judged only the current data because the input data pass once through all the nodes. However, the RNN and LSTM models are used widely in time series analysis, as they simultaneously consider both present and past input data. One disadvantage of the RNN model is that the weight of the initial data decreases as the distance between the input data and the nodes utilizing the data increases. The LSTM model improves on this by continuously updating the weights of the important parts of the input values by using four steps in the model. Compared with the conventional RNN model used in water quality research, the LSTM model used in our study had superior prediction accuracy.

The LSTM model derives its values through four steps: forget, input, update, and output. The forget step chooses which information to discard from the old and new incoming data. This decision is determined by the sigmoid layer in the LSTM cell. At this stage, a value between 0 and 1 is sent, which is the criterion for how much information to pass: if the value is 0, no information is transmitted; if the value is 1, all information is transmitted. In the input step, the model chooses whether to save the new information. As in the previous step, the sigmoid layer determines which values to update. Then, the tanh layer creates new results and adds information from the two layers to update the information. In the update step, the previous cell state is updated using the values determined in the previous step. Finally, the output stage determines the output value (which of the filtered values should be exported through the above process). This structure minimizes the loss of previously learned information and yields results [41].

We conducted comparative analysis using the above model. The detailed hyperparameters in the deep learning model in the instance of the activation function are those of the commonly used rectified linear unit (ReLU) function. In this study, we employed the Adam optimizer, which has the following advantages: it is straightforward to carry out, computationally effective, has minimal memory requirements, is unchangeable to diagonal rescaling of the gradients, and is suited for problems that have large amounts of data and/or parameters. This method is also appropriate for non-stationary purposes and problems with very noisy and/or sparse gradients [42]. The model has three hidden layers, a batch size of 100, and an epoch of 100. With regards to the epoch, values of 100, 300, 500, and 700 were used in the pilot analysis. The LSTM model epoch was set to 100 as it was optimal in terms of time and results. The MLP model epoch was set to 500, which had a lower RMSE value than the other epoch. In addition, a time lag of 1 was specified to construct a model that could predict the results a week in advance.

**Figure 2:**
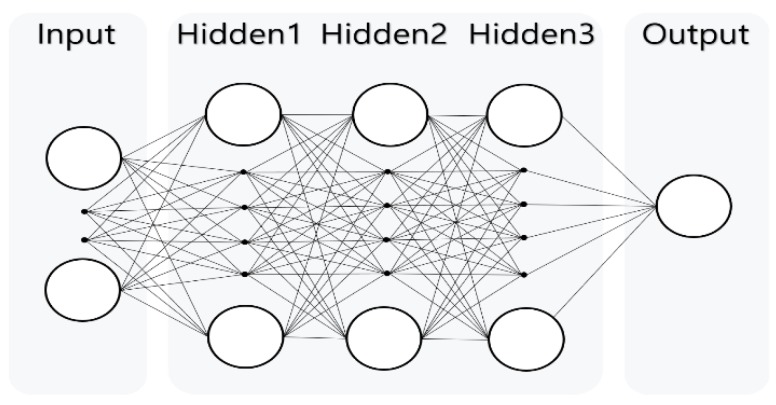
The deep learning model structure using nine input variables, three hidden layers, and one output layer (all fully connected).

Figure 2 shows the structure of the deep learning model, consisting of nine input variables, three hidden layers, and one output layer (all layers were fully connected). The first layers of the hidden layer contained 32 nodes while the second and third layers contained 64 nodes. The same structure was used for the LSTM, RNN, and MLP for proper comparison between the three models.

**Figure 3:**
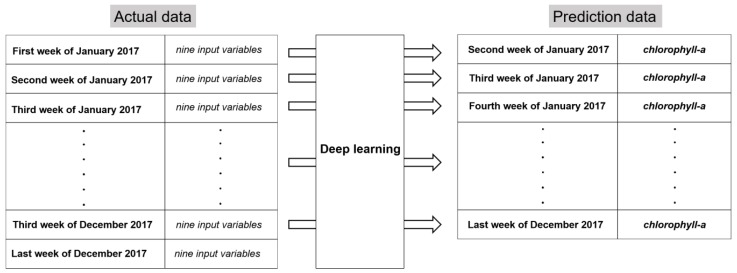
The deep learning prediction process using temporal data.

To compare the prediction values between models, 60% of the data were used as training data, 20% as validation data, and 20% as test data. Figure 3 shows the prediction process, in which predicted chlorophyll-a (*t* point) was a predictor variable for the learning of nine variables in one week (*t*-1 point). One week in advance was chosen as the prediction timescale for algal blooms because it showed the best balance between prediction accuracy and an effective prediction period for preventing algal bloom damage. The test data were used to predict chlorophyll-a and compare with existing data. The RMSE was used as an evaluation index by comparing the predicted value with the actual value:

(2) $$RMSE=\sqrt{mean\left(\Sigma {\left(target-prediction\right)}^{2}\right)}$$

This indicator implies that the lower the value, the closer the predicted data are to the actual value.

### 2.3. Data

#### 2.3.1. Data preprocessing

We obtained data from 16 dammed pools on four major rivers in South Korea. Before using these data, the missing values in the water quality data were replaced with values obtained through linear interpolation. As the date and time of the data differed according to the week and day, we set a standard interval of six days. In addition, the water quality data were measured weekly while the water quantity data were measured daily, so we recalculated the water quantity data to a weekly unit. In this way, weekly algal research data were generated from 27 August 27 2012 to 25 December 2017.

#### 2.3.2. Dependent Variable

After data preprocessing, chlorophyll-a was used as a dependent variable in water quality data from the Ministry of Environment. As a harmful algal bloom progresses, an increase in the number of cyanobacteria cells on the water surface causes the release of harmful toxins. An increase in chlorophyll-a indicates eutrophication of the water as a result of the algal bloom. Along with measuring chlorophyll-a, we intended to measure the number of cyanobacteria cells producing harmful toxins, but this was difficult to analyze owing to a large amount of missing data. Therefore, only chlorophyll-a was used as a dependent variable in this study.

#### 2.3.3. Control and Independent Variables

The data from the branch units of the Ministry of Environment and Ministry of Land, Infrastructure and Transport were collected and used for analysis as control variables and independent variables. The Ministry of Environment data included temperature, pH, conductivity, DO, BOD, COD, T-P, and cyanobacteria. Ministry of Land data included water level, pondage, and amount of precipitation. We then used backward elimination to select the most meaningful variables. Non-significant variables were eliminated sequentially using the *p*-value of *t*. Finally, we selected the following variables: temperature, pH, BOD, COD, DO, cyanobacteria, water level, and pondage (Table 1).

We used nine selection variables and 4464 weekly data points over a period of six years. The amount of DO was used as an indicator of water quality, representing the amount of oxygen pollution. The BOD and COD levels indicated whether the microorganisms needed oxygen to decompose organic matter: the higher these two indices, the more organic matter was present. The water level indicates the surface level of each dammed pool, while pondage indicates the total volume of water.

## 3. Results and Discussion

As shown in Table 2, five variables (temperature, pH, DO, BOD, and COD) were positively correlated with the change in chlorophyll-a, i.e., when chlorophyll-a increased, these parameters also increased. This matches the results of past research showing that an increase in water temperature to 25 °C results in algae growth [27]. The positive regression coefficients for DO, BOD, and COD indicate that, when x increased 1 point, y increased by the coefficient value. With respect to DO, as the algae are photosynthetic and produce oxygen, increasing algae produce more DO [12]. BOD is an important parameter for assessing water pollution; the higher the BOD concentration, the higher the increase in organic matter and chlorophyll-a, as shown by a previous study finding that the correlation between BOD and chlorophyll-a in Ham Nghi Lake (Vietnam) in 2013 produced an R^2^ value of 0.97 [43]. Finally, COD has a strong correlation with BOD and is used when determining the pollution level of a water body because measurements of COD are more accurate than those of BOD, which is significantly affected by carbon assimilation in the presence of algae [44]. Our regression analysis indicated a positive correlation between DO, BOD, and COD, in accordance with previous research.

In contrast, the water level, pondage, and cyanobacteria variables were negatively correlated with changes in chlorophyll-a, i.e., when x increased 1 point, y decreased by the coefficient value. However, changes in water level could lead to a decrease as well as an increase in chlorophyll-a, as a previous study indicated that the prevention of algal blooms was augmented when the rising period of the water level increased [28]. These results confirmed that temperature, cyanobacteria, pH, DO, BOD, and COD were variables affecting chlorophyll-a, in addition to both water quantity and quality.

**Figure 4:**
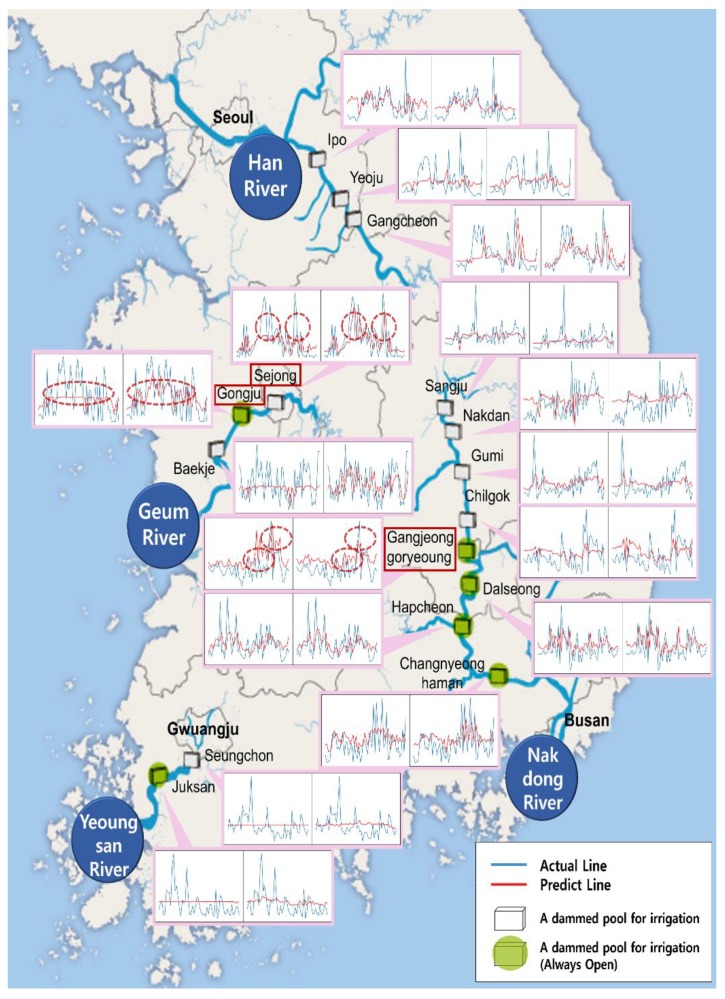
Comparison of MLP (left) and LSTM (right) model results for chlorophyll-a at South Korean dammed pools sites. Blue lines show actual values and red lines show predicted values.

The results of the MLP and LSTM models are shown in Figure 4, in which the chlorophyll-a data were predicted one week in advance. The RMSE value was lower in the LSTM model than in the MLP model, demonstrating the superior accuracy of the LSTM model. Although the MLP’s predictions were superior in some cases, in most instances it did not follow the trend well.

For example, at the Sejongbo site (a dammed pool used for irrigation) on the Geum River, the RMSE value of the MLP model was 33.48, slightly higher than that of the LSTM model. In case of Sejongbo, MLP model predicted values did not increase sharply with a rise in the actual value; however, the LSTM predicted values closely followed the peak points of actual values. A similar result occurred at the nearby Gongjubo site (a dammed pool used for irrigation). In contrast, for Gangjeong goryeoungbo (a dammed pool for irrigation), the MLP model peak point was closer to actual values than that of the LSTM model. However, in other intervals, the predicted values deviated significantly from the actual values, indicated by higher RMSE values than for the LSTM model. At this site, the maximum value of the Y-axis was 40; lower than the other peak. In this case, the MLP model prediction line followed the actual line well. However, for the other two points, the maximum value of the Y-axis was more than 100. In this instance, the MLP model prediction line remained between 60 and 70 and did not follow the trend, while the LSTM model followed the trend well regardless of the size of the value. This limitation of the MLP model appears to explain why its RMSE value was higher than that of the LSTM model.

Table 3 shows the results of RMSE comparisons after executing 100, 300, 500, and 700 epochs. We performed analyses to determine the appropriate epoch for each model. As a result, in the MLP model, the sum of RMSE values seemed to decrease gradually as the epochs increased, but increased again from epochs of 500 or more. For the LSTM model, the RMSE values increased continuously after the 100th epoch. Consequently, for a comparison between the two models, it was necessary to compare the RMSE values in the optimal epoch. Therefore, we selected epoch 100 and 500 for the LSTM and MLP models, respectively. These results suggest that parameter adjustment is required to increase the accuracy of the two models and that increasing the amount of data would also help improve the accuracy. Table 4 shows the results of the RMSE comparison between OLS regression analysis, MLP, RNN, and LSTM.

Our results show that the deep learning models were superior to the OLS linear regression model at most dammed pool sites. We compared the OLS model with each deep learning model. The RMSE values of the MLP was lower than those of OLS at 11 dammed pools, the RNN was lower than OLS at 12 dammed pools, and the LSTM was lower than OLS at 12 dammed pools. The RMSE values of the MLP and RNN were lowest at four and three dammed pools, respectively, while those of the LSTM and OLS were lowest at five dammed pools each. All deep learning models’ RMSE averages were lower than the OLS average; the difference in the RMSE average between the OLS and LSTM model was 1.66. The LSTM deep learning model showed the best prediction performance overall, with the lowest average RSME and lowest individual values at five of the 16 dammed pools, although it performed less well in certain cases. These results demonstrate that the prediction accuracy for algal blooms can be improved through the use of deep learning models, particularly when compared to the commonly used OLS model.

The results of this study support the practicality of using deep learning models to supplement existing models in comparable research contexts. As this is the first attempt, to our knowledge, at predicting algal blooms in Korean rivers using the LSTM model of deep learning, we expect that this research will prompt further attempts to apply and refine these methods.

However, this study has some limitations. We considered the use of cyanobacteria in the prediction of algae blooms, yet there were some missing data values that meant it could not be used, so it became a limiting factor in the accurate prediction of algae blooms. In addition, it is necessary to adjust the parameters in future to obtain higher accuracy; better results will be obtained by adjusting the parameters to determine optimal values for each region. Subsequent studies and/or improved data would be very helpful in overcoming this limitation. In addition, as the predicted results were not always accurate, further research would be useful to better refine the methods employed here.

Furthermore, our results showed the potential for using a deep learning model when it is difficult to apply existing physical prediction models (such as QUAL2E and CE-QUAL-W2) due to a lack of data on the relationships between factors. Applying deep learning methodology to water quality and environment management studies can improve the prediction accuracy by constructing a short-term prediction model for algal blooms. The performance of the LSTM model can achieve better predictions, one week in advance that would enable the implementation of specific and appropriate measures for the prevention or mitigation of algal blooms.

## 4. Conclusions

In this study, we analyzed factors influencing the occurrence and prediction of harmful algal blooms using weekly water quality and quantity data of 16 dammed pools on four major rivers in South Korea. Based on the selected variables, we employed chlorophyll-a as a predictive factor.

Next, we constructed a model using the deep learning method and compared its results with existing analysis methods such as OLS regression analysis to analyze their relative performance. OLS regression and the MLP, RNN, and LSTM deep learning models were investigated by analyzing predictions of chlorophyll-a based on RMSE. The OLS regression model achieved the lowest RMSE value at five of the 16 dammed pools, while the LSTM model was the most accurate overall.

Moreover, the performance of the LSTM model was superior to the MLP and RNN models. In addition, the LSTM model predictions were closer to the actual data than those of the MLP model when variations in chlorophyll-a were large. This implies that the MLP model tended to fail to learn properly when the value of chlorophyll-a increased. The LSTM model, on the other hand, followed the trend line regardless of the range of values. In the comparison between deep learning models, we found that the feedforward method (MLP) performance was worse than that of the recurrent method (RNN and LSTM). In addition, the LSTM model exhibited higher performance than the other models. The algorithms used in the LSTM model were designed to solve the problem of information loss for long-term memory in existing RNN models, which is known to improve predictions by transferring information on previous data as the amount of data grows. Therefore, for water quality data collected daily with continuous data management, predictions could be made more accurate using such “big data.”

## Figures and Tables

**Table 1:** Variables used in model tests and their basic statistical descriptions.

Variable Name	Variable Description	Source	Number of Data	Average	Standard Deviation	Minimum Value	Maximum Value
temperature	water temperature (°C)	Ministry of Environment	4464	17.40	8.16	0.30	34.30
pH	potential of hydrogen		4464	8.00	0.54	5.70	9.70
DO	dissolved oxygen (mg/L)		4464	10.70	2.66	2.20	19.20
BOD	biochemical oxygen demand (mg/L)		4464	2.00	1.26	0.30	9.60
COD	chemical oxygen demand (mg/L)		4464	5.80	1.82	1.80	19.50
cyanobacteria	cyanobacteria cell number		4464	4041	20,695	0	556,740
chlorophyll	chlorophyll-a		4464	23.76	23.15	0.10	177.90
water level	water level (el.m)	Ministry of Land, Infrastructure and Transport	4464	19.49	13.78	1.50	47.52
pondage	pondage (million m^3^)		4464	43.67	30.64	4.829	205.58

Number of Data: 279 × 16 Dammed Pools.

**Table 2:** Multiple linear regression analysis results.

Variable Name	Coefficient	Standard Error	*p* > *t*
temperature	3.262 × 10^−1^	5.918 × 10^−2^	3.74 × 10^−8^ ***
pH	3.218 × 10^−1^	6.706 × 10^−1^	6.31 × 10^−1^
DO	1.466	1.912 × 10^−1^	2.13 × 10^−14^ ***
BOD	2.222	3.455 × 10^−1^	1.39 × 10^−10^ ***
COD	2.580	2.635 × 10^−1^	2 × 10^−16^ ***
cyanobacteria	−6.105 × 10^−5^	1.46 × 10^−5^	2.93 × 10^−5^ ***
water level	−4.891 × 10^−1^	2.85 × 10^−2^	2 × 10^−16^ ***
pondage	−1.260 × 10^−1^	1.076 × 10^−2^	2 × 10^−6^ ***
_cons	−4.18	4.692	3.73 × 10^−1^
*p* > F	2.2 × 10^−16^		
R^2^	0.3032		
adjusted R^2^	0.302		
number of observations	4464		

Significance level: *** *p* < 0.001.

**Table 3:** Results of RMSE comparison by epoch.

Measuring Point	MLP				LSTM			
Epoch	100	300	500	700	100	300	500	700
Ipo	7.84871	8.42762	9.2777	10.7004	7.67382	8.31658	8.73067	9.06951
Yeoju	5.49547	5.6166	6.07492	4.49033	5.61138	5.73774	5.81824	6.13268
Gangcheon	3.64954	3.99566	4.431429	35.5032	3.60946	3.83244	3.86594	3.86588
Sejong	39.6119	35.9101	33.4814	10.3041	30.8273	31.0018	30.9622	31.7447
Gongju	42.7369	35.3198	33.7732	12.2273	31.9164	31.9498	32.1146	33.1228
Baekje	36.477	27.7607	26.5994	12.7383	27.3673	27.1477	27.1138	27.0187
Sangju	14.7071	14.0804	14.0761	26.0294	14.4853	14.4771	14.2902	14.1571
Nakdan	9.96028	10.4088	10.5436	14.0869	9.84722	9.50639	10.137	10.0699
Gumi	11.0802	10.2963	10.1364	32.6677	10.5159	10.2251	10.0779	10.2275
Chilgok	10.3898	10.2936	9.80221	29.1884	11.0027	10.5753	10.2638	10.204
Gangjeong goryeoung	9.2862	8.20598	9.2837	5.8793	7.85588	8.00324	8.78411	8.99846
Dalseong	10.2755	11.3021	11.7977	9.91085	12.6251	12.7122	13.1197	13.4175
Hapcheon	15.0435	13.9717	13.9468	27.4545	14.1113	13.9893	14.1398	13.9613
Changnyeong haman	12.1064	12.2411	12.0053	12.288	13.2302	12.6724	12.501	12.416
Seungchon	36.0572	29.4183	29.0971	9.44017	30.4663	36.1613	37.7719	40.2004
Juksan	29.7646	28.017	28.4197	14.114	26.3498	26.865	26.9653	26.7871
Sum of RMSE	**294.4903**	**265.2658**	**262.7467**	**267.0229**	**257.4954**	**263.1734**	**266.6562**	**271.3935**

**Table 4:** Results of RMSE comparison.

Measuring Point	OLS	MLP	RNN	LSTM
Ipo	13.21	9.28	7.93	7.67
Yeoju	9.13	6.07	5.60	5.61
Gangcheon	6.50	4.43	3.58	3.61
Sejong	29.78	33.48	30.42	30.83
Gongju	32.30	33.77	32.08	31.92
Baekje	25.30	26.60	25.95	27.37
Sangju	10.18	14.08	14.37	14.49
Nakdan	11.88	10.54	9.34	9.85
Gumi	13.32	10.14	10.26	10.52
Chilgok	11.82	9.80	10.55	11.00
Gangjeong goryeoung	10.02	9.28	8.11	7.86
Dalseong	19.63	11.80	13.24	12.63
Hapcheon	14.87	13.95	14.35	14.11
Changnyeong haman	19.40	12.01	12.83	13.23
Seungchon	34.24	29.10	33.25	30.47
Juksan	22.44	28.42	26.22	26.35
RMSE average	17.75	16.42	16.13	16.09

## References

[1] Kahru M., Mitchell B.G. (2008). Ocean Color Reveals Increased Blooms in Various Parts of the World. EOS.

[2] Clark J.M., Schaeffer B.A., Darling J.A., Urquhart E.A., Johnston J.M., Ignatius A.R., Myer M.H., Loftin K.A., Werdell P.J., Stumpf R.P. (2017). Satellite monitoring of cyanobacterial harmful algal bloom frequency in recreational waters and drinking water sources. Ecol. Indic..

[3] Falconer I.R., Burch M.D., Steffensen D.A., Choice M., Coverdale O.R. (1994). Toxicity of the blue-green alga (cyanobacterium) *Microcystis aeruginosa* in drinking water to growing pigs, as an animal model for human injury and risk assessment. Environ. Toxicol. Water Qual..

[4] Heil C.A., Glibert P.M., Fan C. (2005). *Prorocentrum minimum* (Pavillard) Schiller: A review of a harmful algal bloom species of growing worldwide importance. Harmful Algae.

[5] Svendsen M.B.S., Andersen N.R., Hansen P.J., Steffensen J.F. (2018). Effects of Harmful Algal Blooms on Fish: Insights from *Prymnesium parvum*. Fishes.

[6] Backer L.C., Manassaram-Baptiste D., LePrell R., Bolton B. (2015). Cyanobacteria and Algae Blooms: Review of Health and Environmental Data from the Harmful Algal Bloom-Related Illness Surveillance System (HABISS) 2007–2011. Toxins.

[7] Dodds W.K., Bouska W.W., Eitzmann J.L., Pilger T.J., Pitts K.L., Riley A.J., Schloesser J.T., Thornbrugh D.J. (2009). Eutrophication of U.S. Freshwaters: Analysis of Potential Economic Damages. Environ. Sci. Techonol..

[8] McPartlin D.A., Loftus J.H., Crawley A.S., Silke J., Murphy C.S., O’Kennedy R.J. (2017). Biosensors for the monitoring of harmful algal blooms. Curr. Opin. Biotechnol..

[9] Jeong D.I., Ryu D.H., Na E.H., Song S.H., Hwang H.S., Kim E.K., Kim H.K., Kim S.Y. (2006). Hydraulic and Water Quality Modelling.

[10] McAvoy D.C., Masscheleyn P., Peng C., Morrall S.W., Casilla A.B., Lim J.M.U., Gregorio E.G. (2003). Risk assessment approach for untreated wastewater using the QUAL2E water quality model. Chemosphere.

[11] Zhang Z., Sun B., Johnson B.E. (2015). Integration of a benthic sediment diagenesis module into the two dimensional hydrodynamic and water quality model—CE-QUAL-W2. Ecol. Model..

[12] Chae B., Koo J., Lee S., Kwon J., Kong S., Song G. (2017). Development of Prediction Model for Machine Learning Based Algal Bloom.

[13] Najafabadi M.M., Villanustre F., Khoshgoftaar T.M., Seliya N., Wald R., Muharemagic E. (2015). Deep learning applications and challenges in big data analytics. J. Big Data.

[14] LeCun Y., Bengio Y., Hinton G. (2015). Deep Learning. Nature.

[15] Song Q., Zhao M.-R., Zhou X.-H., Xue Y., Zheng Y.-J. (2017). Predicting gastrointestinal infection morbidity based on environmental pollutants: Deep learning versus traditional models. Ecol. Indic..

[16] Li X., Peng L., Yao X., Cui S., Hu Y., You C., Chi T. (2017). Long short-term memory neural network for air pollutant concentration predictions: Method development and evaluation. Environ. Pollut..

[17] Wua C.L., Chau K.W. (2011). Rainfall–runoff modeling using artificial neural network coupled with singular spectrum analysis. J. Hydrol..

[18] Felder M., Kaifel A., Graves A. (2010). Wind power prediction using mixture density recurrent neural networks. Proceedings of the European Wind Energy Conference and Exhibition.

[19] Kim B., Choi K., Kim C., Lee U.-H., Kim Y.-H. (2000). Effects of the summer monsoon on the distribution and loading of organic carbon in a deep reservoir, Lake Soyang, Korea. Water Res..

[20] Ha K., Cho E.-A., Kim H.-W., Joo G.-J. (1999). Microcystis bloom formation in the lower Nakdong River, South Korea: Importance of hydrodynamics and nutrient loading. Mar. Freshw. Res..

[21] Kim S.-G., Rhee S.-K., Ahn C.-Y., Ko S.-R., Choi G.-G., Bae J.-W., Park Y.-H., Oh H.-M. (2006). Determination of Cyanobacterial Diversity during Algal Blooms in Daechung Reservoir, Korea, on the Basis of cpcBA Intergenic Spacer Region Analysis. Appl. Environ. Microbiol..

[22] Gers F.A., Schmidhuber J., Cummins F. (1999). Learning to forget: Continual prediction with LSTM. ICANN.

[23] Kalchbrenner N., Danihelka I., Graves A. (2016). Grid Long Short-Term Memory. arXiv.

[24] Anderson D.M., Glibert P.M., Burkholder J.M. (2002). Harmful algal blooms and eutrophication: Nutrient sources, composition, and consequences. Estuaries.

[25] Paerl H.W., Paul V.J. (2012). Climate change: Links to global expansion of harmful cyanobacteria. Water Res..

[26] Paerl H.W., Fulton R.S., Moisander P.H., Dyble J. (2001). Harmful Freshwater Algal Blooms, with an Emphasis on Cyanobacteria. Sci. World J..

[27] Davis T.W., Berry D.L., Boyer G.L., Gobler C.J. (2009). The effects of temperature and nutrients on the growth and dynamics of toxic and non-toxic strains of Microcystis during cyanobacteria blooms. Harmful Algae.

[28] Ji D., Wells S.A., Yang Z., Liu D., Huang Y., Ma J., Berger C.J. (2017). Impacts of water level rise on algal bloom prevention in the tributary of Three Gorges Reservoir, China. Ecol. Eng..

[29] AMichalak M., Anderson E.J., Beletsky D., Boland S., Bosch N.S., Bridgeman T.B., Chaffin J.D., Cho K., Confesor R., Daloğlu I. (2013). Record-setting algal bloom in Lake Erie caused by agricultural and meteorological trends consistent with expected future conditions. PNAS.

[30] Li F., Zhang H., Zhu Y., Xiao Y., Chen L. (2013). Effect of flow velocity on phytoplankton biomass and composition in a freshwater lake. Sci. Total Environ..

[31] Boyer J.N., Kelble C.R., Ortner P.B., Rudnick D.T. (2009). Phytoplankton bloom status: Chlorophyll a biomass as an indicator of water quality condition in the southern estuaries of Florida, USA. Ecol. Indic..

[32] Li J., Zhang J., Liu L., Fan Y., Li L., Yang Y., Lu Z., Zhang X. (2015). Annual periodicity in planktonic bacterial and archaeal community composition of eutrophic Lake Taihu. Sci. Rep..

[33] Wang H., Gao Y., Xu Z., Xu W. (2011). Elman’s Recurrent Neural Network Applied to Forecasting the Quality of Water Diversion in the Water Source of Lake Taihu. Energy Procedia.

[34] Meloun M., Militký J. (2001). Detection of single influential points in OLS regression model building. Anal. Chim. Acta.

[35] Paerl H.W., Xu H., McCarthy M.J., Zhu G., Qin B., Li Y., Gardner W.S. (2011). Controlling harmful cyanobacterial blooms in a hyper-eutrophic lake (Lake Taihu, China): The need for a dual nutrient (N & P) management strategy. Water Res..

[36] Chai T., Draxler R.R. (2014). Root mean square error (RMSE) or mean absolute error (MAE)?—Arguments against avoiding RMSE in the literature. Geosci. Model Dev..

[37] Olyaie E., Banejad H., Chau K.-W., Melesse A.M. (2015). A comparison of various artificial intelligence approaches performance for estimating suspended sediment load of river systems: A case study in United States. Environ. Monit. Assess..

[38] Taormina R., Chau K.-W., Sivakumar B. (2015). Neural network river forecasting through baseflow separation and binary-coded swarm optimization. J. Hydrol..

[39] Koskela T., Lehtokangas M., Saarinen J., Kaski K. Time Series Prediction with Multilayer Perceptron, FIR and Elman Neural Networks. Proceedings of the World Congress on Neural Networks.

[40] Goh A.T.C. (1995). Back-propagation neural networks for modeling complex systems. Artif. Intell. Eng..

[41] Greff K., Srivastava R.K., Steunebrink B.R., Schmidhuber J. (2016). LSTM: A Search Space Odyssey. IEEE.

[42] Kingma D.P., Ba J.L. (2015). ADAM: A Method for Stochastic Optimization. arXiv.

[43] Phu S.T.P. (2014). Research on the Correlation between Chlorophyll-a and Organic Matter BOD, COD, Phosphorus, and Total Nitrogen in Stagnant Lake Basins. Sustain. Living Environ. Risks.

[44] Kumar A., Dhall P., Kumar R. (2010). Redefining BOD: COD ratio of pulp mill industrial wastewaters in BOD analysis by formulating a specific microbial seed. Int. Biodeterior. Biodegrad..

